# Irradiation alters extracellular vesicle microRNA load in the serum of patients with leukaemia

**DOI:** 10.1007/s00066-024-02307-6

**Published:** 2024-09-26

**Authors:** Stephanie Hehlgans, Denise Eckert, Daniel Martin, Katalin Lumniczky, Gesine Bug, Claus Rödel, Franz Rödel

**Affiliations:** 1 Department of Radiotherapy and Oncology, University Hospital Frankfurt, Goethe University Frankfurt, Theodor-Stern-Kai 7, 60590 Frankfurt am Main, Germany; 2 DKFZ-Hector Cancer Institute at the University Medical Center Mannheim, Department of Personalized Oncology, University Hospital Mannheim, Medical Faculty Mannheim, University of Heidelberg, Mannheim, Germany; 3 Frankfurt Cancer Institute, Goethe University Frankfurt, Theodor-Stern-Kai 7, 60590 Frankfurt, Germany; 4 German Cancer Consortium (DKTK), Partner Site Frankfurt/Mainz and German Cancer Research Center (DKFZ), Im Neuenheimer Feld 280, 69120 Heidelberg, Germany; 5 National Center for Public Health and Pharmacy, Department of Radiobiology and Radiohygiene, Unit of Radiation Medicine, Budapest, Hungary; 6 Department of Medicine II, Hematology and Oncology, University Hospital Frankfurt, Goethe University Frankfurt, Theodor-Stern-Kai 7, 60590 Frankfurt am Main, Germany; 7 Division of Personalized Medical Oncology (A420), German Cancer Research Center (DKFZ), German Center for Lung Research (DZL), 69120 Heidelberg, Germany

**Keywords:** Acute lymphoblastic leukaemia, Acute myeloid leukaemia, Extracellular vesicles, Biomarker, Irradiation exposure, MiRNA

## Abstract

**Purpose:**

Recent data suggest an impact of extracellular vesicles (EVs) and their micro(mi)RNA cargo on cell-cell interactions to contribute to pathophysiology of leukaemia and radiation response. Here, we investigated differential miRNA cargo of EVs from serum derived from patients with leukaemia (*n* = 11) before and after total body irradiation with 2 × 2 Gy as compared to healthy donors (*n* = 6).

**Methods:**

RNA was isolated from EVs and subjected to next generation sequencing of miRNAs. Analysis of sequencing data was performed with miRDeep29 software and differentially expressed miRNAs were filtered using R package edgeR10,11. Signaling pathways were identified using Kyoto Encyclopedia of Genes and Genomes database (KEGG) pathway analysis.

**Results:**

Flow cytometric and Western blot analyses confirmed the presence of characteristic EV markers TSG-101, CD‑9 and CD-81. miRNA sequencing revealed a differential cargo in serum of patients with leukaemia in comparison to healthy donors with 23 significantly upregulated and 16 downregulated miRNAs affecting hedgehog, estrogen, glutathione metabolism and peroxisome proliferator-activated receptor (PPAR) signaling pathways amongst others. Whole body irradiation of patients with leukaemia significantly increased 11 miRNAs, involved in cell cycle regulation and platinum drug resistance, and decreased 15 miRNAs, contributing to apoptosis or cytokine-receptor interactions.

**Conclusion:**

As compared to healthy controls and following irradiation, we have identified differentially regulated miRNAs in serum-derived EVs from patients with leukaemia that may serve as possible biomarkers of leukaemic disease and treatment and radiation exposure.

**Supplementary Information:**

The online version of this article (10.1007/s00066-024-02307-6) contains supplementary material, which is available to authorized users.

## Introduction

Acute myeloid and lymphoblastic leukaemias are malignant diseases of the hematopoietic system which initially require intensive chemotherapy for curative intent [1]. Initial treatment is followed by consolidation chemotherapy or an allogeneic hematopoietic cell transplantation (HCT). By this, consolidation chemotherapy consisting of dexamethasone, vincristine, and doxorubicin, followed by cyclophosphamide, Ara‑C, and 6‑thioguanine is given in patients with ALL [2], while patients with AML are consolidated with intermediate or high doses of cytarabine (HDAC) and Fms-like Tyrosine Kinase 3 (FLT3)-inhibitors in patients with FLT3-mutations [3]. As leukaemic cells are exquisitely sensitive to irradiation, total body irradiation (TBI) is routinely administered in combination with chemotherapy as conditioning prior to HCT in patients with ALL and less often with AML. Leukaemic relapse remains a frequent and serious problem [4].

Extracellular vesicles (EVs) function as a key player in intracellular communication. They cover a group of various membrane-coated bodies released by several cell types and contain a variety of biological cargo such as lipids, proteins, and nucleic acids, including microRNAs (miRNAs) [5, 6]. EVs can be grouped into exosomes (40–100 nm), microvesicles (20–1000 nm) and apoptotic bodies (up to 5000 nm) based on their biogenesis and size distribution. The molecular cargo is protected from degradation by enzymes due to the packaging vesicles and, thus, can be delivered to the recipient cell in an active form to modulate signaling pathways. Recent data suggest that EVs are involved in leukaemogenesis and in the pathophysiology of leukaemia by modulating cancer cell interaction with the bone marrow microenvironment, mainly by affecting gene expression in the stem cell compartment [7], and by altering apoptosis, tumor angiogenesis, immune escape and therapy resistance [8]. Further, EVs may contain potential biomarkers and their analysis from “liquid biopsies” is emerging as a valuable approach for the diagnosis, prognosis and therapeutic monitoring of cancer patients [8, 9].

miRNAs are evolutionarily conserved short single-stranded noncoding RNA molecules (20–24 nucleotides) that play a pivotal role in posttranscriptional regulation of messenger RNA targets of gene expression [10]. Thereby, miRNAs are involved in the regulation of more than 60% of mammalian protein-coding genes [11] and cover important roles in multiple biological processes including cell differentiation, inflammation, apoptosis, cell cycle regulation, stress response, cancer development, and metastasis [12]. In addition, in vitro studies using peripheral blood mononuclear cells revealed radiation-induced changes to occur in the protein and miRNA cargo of EVs indicating systemic communication pathways between irradiated and non-irradiated cells [13]. Moreover, in vivo studies demonstrated that EVs derived from the bone marrow of irradiated mice can mediate bystander effects in the hematopoietic system of EV recipients, thus showing a change in a panel of eight differentially expressed miRNAs with a predicted involvement in pathways related to DNA damage repair, and immune system regulation [14].

As EVs can be isolated from all body fluids (e.g. blood, urine) and from tissue of patients, they cover a potential source for biomarker development [9]. Individual protein and/or miRNA fingerprints, for example in peripheral blood EVs, may provide an indication of disease stages, subclinical relapse or minimal residual disease, and treatment response to radiation exposure. In fact, the first EV-derived miRNA (miR155) is already being tested as a potential biomarker in chronic lymphocytic and acute myeloid leukaemia [15].

In the present study, we aimed to identify miRNAs as biomarkers for leukaemia and leukaemic chemotherapeutic treatment and radiation exposure in serum-derived EVs from patients with leukaemia treated with whole body irradiation.

## Materials and methods

### Patient characteristics

In this study, *n* = 7 patients with AML (median age: 48.9 years, range 21–67 years, 5 male, 2 female) and *n* = 4 patients with ALL (median age: 47.8 years, range 22–59 years, 1 male, 3 female) were enrolled, while *n* = 6 healthy donors (median age: 41.8 years, range 30–56 years, 2 male, 4 female) served as controls. Patients were treated with total body irradiation (TBI) as part of the conditioning regimen prior to an allogeneic hematopoietic cell transplantation. In the patient cohort, pre-transplant minimal residual disease (MRD) or refractory disease was detected in *n* = 7 patients (64%), *n* = 1 patient (9%) was of unknown status and *n* = 3 patients (27%) were MRD negative. The trial was approved by the Institutional Ethics Committee of the University Hospital Frankfurt am Main (protocol code 15/18) and was conducted in accordance with the Declaration of Helsinki. All patients have signed an informed consent.

### Blood collection from patients and healthy donors

Peripheral blood was collected from patients with leukaemia via port catheter using 7.5 ml Serum/CAT tubes (Sarstedt, Nümbrecht, Germany) at the Department of Medicine II, Hematology and Oncology, University Hospital, Goethe University Frankfurt am Main. Blood samples were taken before TBI by a photon beam linear accelerator (Synergy, Elekta, Crowley, UK) with doses of 2 × 2 Gy per day for a total dose of 4 Gy at the Department of Radiotherapy and Oncology with an 8‑hour interval between doses and at 24 h after irradiation. All but one patient received chemotherapy between nine and two days prior to TBI. Peripheral blood of healthy donors served as a control.

### Isolation of EVs from serum

EVs were isolated from peripheral blood using an ultracentrifugation workflow. In detail, blood from patients with leukaemia or healthy donors was allowed to clot for 30 min at RT and centrifuged at 1000 × g for 15 min to obtain serum. Subsequently, serum was centrifuged at 3000 × g for 15 min at room temperature and supernatant was centrifuged at 30,000 × g for 30 min. Next, supernatant was filtered through a 0.2 µm filter (Carl Roth, Karlsruhe, Germany) and centrifuged at 100,000 × g in an ultracentrifuge (Beckman Coulter, Krefeld, Germany) for two hours. Finally, the pellet was resuspended in 150 µL PBS (Thermo Fisher Scientific, Darmstadt, Germany) and EVs were stored at −80 °C for further analyses.

### Quantification of protein concentration of EVs using BCA assay

To determine the protein concentration of isolated EVs, the Micro BCATM Protein-Assay-Kit (Thermo Fischer Scientific, #23235,) was used according to the manufacturer’s protocol. In brief, 150 µl of standard and 149 µl of ddH2O + 1 µl of isolated EVs were pipetted in triplicates in a 96-well plate (Greiner Bio-One, Frickenhausen, Germany), 150 µl Micro BCATM assay working reagent were added, and the plates were incubated for 2 h at 37 °C. Finally, optical densities were measured at 562 nm using a 96-well microplate reader (Infinite M200 Pro, TECAN, Männedorf, Switzerland) and concentrations were determined according to standard protein dilutions.

### Characterization of EVs by Western immunoblotting

To characterize purified EVs, Western blot analysis was performed. 100 µg of EVs were lysed in modified radioimmunoprecipitation assay buffer (RIPA) as previously described [16], and incubated for 30 min on ice, followed by a heating step for 5 min at 99 °C. The lysed EVs were subjected to SDS-polyacrylamide gel electrophoresis (SDS-PAGE) and transferred to a Nitrocellulose membrane (GE Healthcare, Munich, Germany) using a semidry blotter unit (GE Healthcare). Membranes were incubated over night at 4 °C with the following primary antibodies: anti-Calnexin rabbit (rb) (Abcam, Cambridge, UK, #ab22595, 1:1000, 95 kDa), anti-TSG101 rb (Abcam, #ab125011, 1:1000, 45 kDa), anti-CD9 rb (Abcam, #ab92726, 1:1000, 25 kDa). After incubation with a secondary antibody (goat-anti-rabbit-IgG HRP, Biozol, Eching, Germany, #4050-05, 1:1000) for 1 h at room temperature, detection was accomplished using a Pierce ECL Western blotting substrate (Thermo Fischer Scientific) and the Odyssey Fc imaging system (LI-COR Biotechnology, Bad Homburg, Germany) for documentation.

### Validation of EVs by flow cytometric analysis

To further verify isolated EVs by flow cytometry, 40 µg of EVs were stained with CD81 (APC-conjugated, clone 5A6, BioLegend, Amsterdam, The Netherlands, #349509) and CD9 (FITC-conjugated, Thermo Fisher Scientific, clone SN4 C3-3A2, #11-0098-42) for 1 h at 4 °C. Next, EVs were washed with PBS and precipitated at 100,000 × g for 70 min at 4 °C. The pellet was resuspended in 150 µl PBS and measured using flow cytometry (CytoFlex S, Beckman Coulter). The violet side scatter (V-SSC) was used as described in Brittain et al. 2019 [17]. For correct gating of EVs, Gigamix beads (Beckman Coulter) were used to determine the size standard for EVs. In a V-SSC-A/FSC‑A dot plot EVs/beads with a size around 100 to 900 nm were determined. Subsequently, percentages of FITC-positive (CD9) or APC-positive (CD81) EVs/beads were identified in a V-SSC-A/FITC or V‑SSC-A/APC dot plot.

### RNA isolation from serum-derived EVs

Total mRNA, including miRNA, was isolated from EVs with an RNeasy Mini Kit (Qiagen, Hilden, Germany) and RNA content was quantified by photospectroscopic analyses (Infinite M200 Pro, TECAN).

### Next generation sequencing of EV miRNA cargo

Total RNA was subjected to next generation sequencing (NGS) at Arraystar Inc. (Rockville, MD, USA) using an Illumina NextSeq 500 system. Briefly, samples covering in total six samples of healthy donors and 11 samples from patients prior to and after irradiation were transferred to the company and sequenced. The sequencing quality score revealed sufficient quality for subsequent data analysis in all samples. After quality control, the reads were 3′-adaptor trimmed and filtered ≤ 15 bp reads with cutadapt software. The trimmed reads were aligned to reference genome with bowtie software. Data analysis after trimming was performed with the software miRDeep29 to quantify known miRNA and predict novel miRNAs. Additionally, differentially expressed miRNAs were filtered using R package edgeR10,11. Hierarchical clustering and miRNA target prediction was performed by targetscan. Pathway analysis for the miRNA target mRNAs was performed based on the on the top 10 differentially expressed miRNAs using the latest Kyoto Encyclopedia of Genes and Genomes (KEGG) database [18]. This analysis allows to determine whether the identified miRNA target genes are enriched in specific biological pathways. The *p* values calculated by Fisher’s exact test are thereby used to estimate the statistical significance of pathway enrichment between the two groups compared. The KEGG database resource was developed to better understand functions of biological systems, including cells and organs, based on information on the molecular level, e.g., large-scale datasets from NGS sequencing approaches or other high-throughput technologies [18]. A schematic workflow of sequencing data analysis (Arraystar Inc. Rockville, USA) is shown in Supplementary Figure S1. Venn diagrams to identify similarities between the differentially expressed miRNAs were created using the InteractiVenn software (www.interactivenn.net, [19]).

### Statistical evaluation

EdgeR was used to evaluate differentially expressed miRNAs. The sequencing raw data were fitted to negative binomial model using the quantile-adjusted conditional maximum likelihood (qCML) method as implemented in EdgeR. The differentially expressed genes were then tested by exact test using the model (for details see [20]). The *p* value is the f‑statistic *p* value, the *q* value (false discovery rate, FDR) comprises the FDR-adjusted *p* value. A *p* value less or equal to 0.05 and a q value less or equal to 1 were considered statistically significant. For KEGG pathway analysis, *p* values calculated by Fisher’s exact test were used to estimate the statistical significance of the enrichment of the pathways between the two groups.

## Results

### Characterization of EVs from patients and healthy donors

**Fig. 1:**
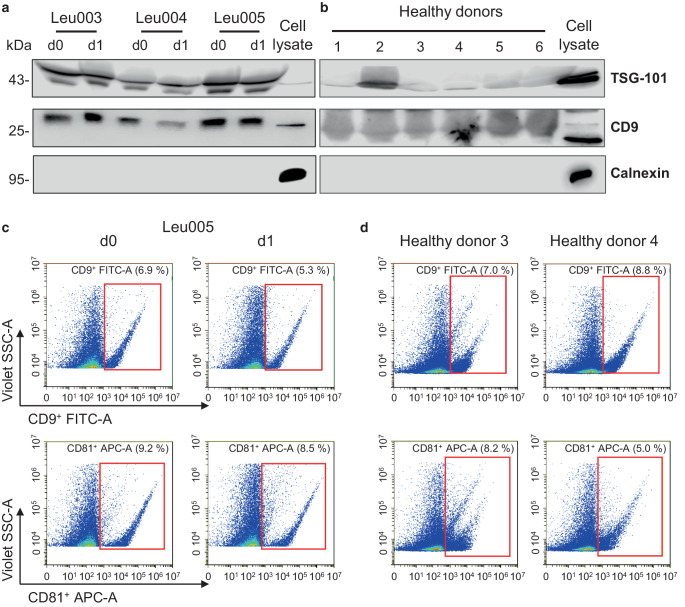
Detection of EV markers in EVs from patients and healthy donors. Representative Western blots from patients (**a**) and healthy donors (**b**) show detection of TSG-101 and CD9, but not Calnexin. Flow cytometric analyses of CD9 and CD81 positive events in one patient before (d0) and after (d1) irradiation (**c**) and two healthy donors (**d**)

To characterize EVs derived from patients with leukaemia and healthy donors, Western blot analyses were performed using common EV markers such as CD9 and TSG-101 and the endoplasmic reticulum (ER) membrane marker Calnexin as a negative control. Representative immunoblots from three patients with leukaemia and six healthy donors are shown in Fig. 1a,b. Both groups expressed the EV markers CD9 and TSG-101. As expected, Calnexin detection was restricted to control cell lysate (Fig. 1a,b). These findings were confirmed by flow cytometric analyses. The EV markers CD81 and CD9 were detected in both, isolated EVs from patients and healthy donors (Fig. 1c,d).

### Comparison of miRNA load in extracellular vesicles of patients with leukaemia vs. healthy donors

**Fig. 2:**
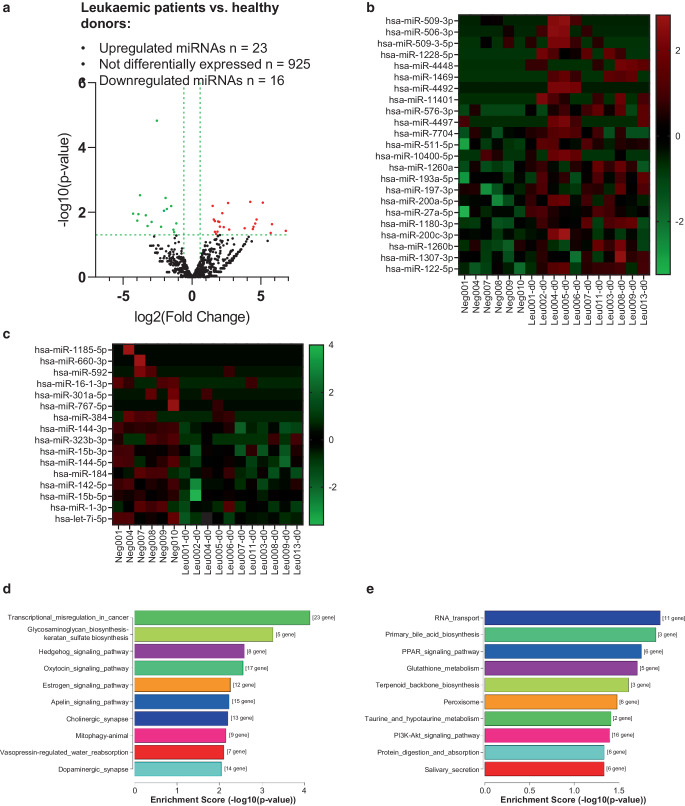
Differentially expressed miRNAs of EVs derived from serum of patients with leukaemia before irradiation vs. healthy donors. **a** Volcano plot; X axis: log2 transformed fold change; Y axis: −log10 transformed *p*-values. **b** Heatmap of upregulated and **c** heatmap of downregulated miRNAs in EVs from serum of patients with leukaemia. **d** Top 10 significant Kyoto Encyclopedia of Genes and Genomes database (KEGG) pathways affected by upregulated miRNAs of patients with leukaemia vs. healthy donors, ordered from top to bottom by *p* value, with the most significant pathway on the top. **e** Top 10 significant KEGG pathways affected by downregulated miRNAs of patients with leukaemia vs. healthy donors, ordered from top to bottom by *p*-value

To investigate, whether serum EVs derived from peripheral blood of patients with leukaemia prior to irradiation contain a differentially regulated set of miRNAs potentially valuable as leukaemic and treatment markers, we first compared miRNA NGS data from four ALL and seven AML patients with findings from six healthy controls. This comparison yielded 23 significantly upregulated and 16 significantly downregulated miRNAs (Fig. 2; Table 1), while 925 miRNAs were not affected.

**Table 1:** Significantly up- (*n* = 23) and downregulated (*n* = 16) miRNAs in EVs from serum of patients with leukaemia vs. healthy volunteers

Mature miRNA ID	Log2FC	Fold Change	Log2(CPM) leukaemia	Log2(CPM) controls	*p* value	q value
*Upregulated (n* *=* *23)*						
Hsa-miR-509-3p	6.811	112.299	8.523	1.712	0.037	0.978
Hsa-miR-506-3p	5.800	55.706	7.080	1.280	0.023	0.905
Hsa-miR-509-3-5p	5.708	52.287	9.635	3.927	0.043	1.000
**Hsa-miR-1228-5p**	5.144	35.350	5.161	0.017	0.005	0.780
Hsa-miR-4448	4.689	25.795	4.706	0.017	0.017	0.876
*Hsa-miR-1469*	4.590	24.080	4.607	0.017	0.022	0.898
Hsa-miR-4492	4.465	22.080	4.482	0.017	0.028	0.922
Hsa-miR-11401	4.413	21.310	4.431	0.017	0.033	0.978
**Hsa-miR-576-3p**	4.234	18.824	5.827	1.593	0.005	0.780
Hsa-miR-4497	3.819	14.111	5.448	1.629	0.031	0.966
Hsa-miR-7704	2.702	6.509	6.139	3.437	0.034	0.978
Hsa-miR-511-5p	2.615	6.127	7.579	4.964	0.005	0.780
Hsa-miR-10400-5p	2.307	4.949	5.821	3.513	0.029	0.922
**Hsa-miR-1260a**	2.203	4.604	8.135	5.932	0.011	0.785
Hsa-miR-193a-5p	2.022	4.062	6.965	4.942	0.028	0.922
Hsa-miR-197-3p	1.983	3.953	8.275	6.292	0.020	0.876
Hsa-miR-200a-5p	1.897	3.724	8.409	6.512	0.019	0.876
Hsa-miR-27a-5p	1.854	3.616	7.299	5.445	0.040	0.987
Hsa-miR-1180-3p	1.749	3.361	7.444	5.695	0.046	1.000
Hsa-miR-200c-3p	1.663	3.167	10.900	9.237	0.019	0.876
Hsa-miR-1260b	1.661	3.162	8.439	6.778	0.040	0.987
Hsa-miR-1307-3p	1.583	2.996	10.589	9.006	0.017	0.876
**Hsa-miR-122-5p**	1.501	2.830	16.200	14.699	0.006	0.780
*Downregulated (n* *=* *16)*						
Hsa-miR-1185-5p	−4.275	0.052	0.017	4.292	0.011	0.785
Hsa-miR-660-3p	−3.974	0.064	0.017	3.992	0.018	0.876
Hsa-miR-592	−3.893	0.067	0.783	4.677	0.011	0.785
**Hsa-miR-16-1-3p**	−3.777	0.073	1.814	5.592	0.003	0.780
Hsa-miR-301a-5p	−3.359	0.097	1.311	4.670	0.012	0.795
Hsa-miR-767-5p	−3.223	0.107	0.575	3.798	0.026	0.922
Hsa-miR-384	−2.935	0.131	1.870	4.806	0.020	0.876
**Hsa-miR-144-3p**	−2.548	0.171	8.372	10.919	0.000	0.014
Hsa-miR-323b-3p	−2.244	0.211	3.645	5.889	0.028	0.922
**Hsa-miR-15b-3p**	−2.039	0.243	5.391	7.430	0.009	0.785
**Hsa-miR-144-5p**	−1.914	0.265	7.295	9.209	0.004	0.780
**Hsa-miR-184**	−1.828	0.282	11.775	13.603	0.008	0.785
**Hsa-miR-142-5p**	−1.510	0.351	10.081	11.591	0.006	0.780
Hsa-miR-15b-5p	−1.347	0.393	7.939	9.285	0.035	0.978
Hsa-miR-1-3p	−1.303	0.405	13.061	14.364	0.041	0.994
**Hsa-let-7i-5p**	−1.138	0.454	14.440	15.578	0.022	0.898

*p* value cut-off ≤ 0.05, *q* value cut-off ≤ 1. *Mature miRNA ID* ID of mature miRNA; *CPM* counts per million reads; *log2FC* difference in mean between two groups (Test_CPM vs. Control_CPM) of negative binomial random variables; Fold change, *2*^*test_CPM-control_CPM*^ Log2(CPM), average of log CPM scaled by qCML methods: log2(CPM) of miRNA in respective groups; *p value* F-statistic *p* value; *q value* FDR adjusted *p* value. miRNAs up- or downregulated in the combined cohort and also in AML and ALL patients are highlighted in bold

The volcano plot of differentially expressed miRNAs in patients with AML and ALL is shown in Fig. 2a, upregulated miRNAs are presented as a heatmap in Fig. 2b, and downregulated miRNAs are given in Fig. 2c. To unravel which cellular pathways will be affected by the differentially regulated miRNAs, KEGG pathway analysis were performed. The top 10 significant pathways altered by upregulated miRNAs are depicted in Fig. 2d and cover “Transcriptional misregulation in cancer”, “Glycosaminoglycan-Biosynthesis-keratan sulfate biosynthesis” and “Hedgehog signaling pathway”. Pathways most affected by downregulated miRNAs include “RNA transport”, “PPAR signaling”, and “Glutathione metabolism” (Fig. 2e).

### Comparison of miRNA load in extracellular vesicles of patients with leukaemia before and after radiation therapy

**Fig. 3:**
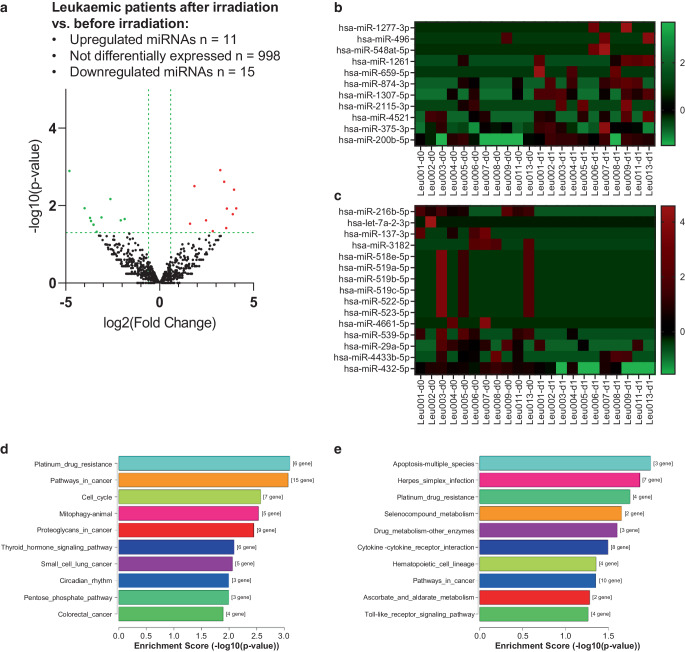
Differentially expressed miRNAs of EVs derived from serum of patients with leukaemia after whole-body irradiation vs. before irradiation. **a** Volcano plot, **b** heatmap of upregulated and **c** heatmap of downregulated miRNAs in EVs from serum of patients with leukaemia after and before irradiation. **d** Top 10 KEGG pathways affected by upregulated miRNAs or **e** downregulated miRNAs from irradiated vs. non-irradiated patients with leukaemia

Next, we analyzed the miRNA load of serum-derived EVs from patients with leukaemia before (d0) and after a 2 × 2 Gy TBI (d1) preceding stem cell transplantation to explore in more detail alterations of EV miRNA markers following radiation exposure. By this, radiation significantly upregulated 11 miRNAs and downregulated 15 miRNAs, whereas 998 miRNAs were not differentially expressed (Fig. 3a–c and Table 2). Radiation exposure-induced miRNAs most significantly affected “Platinum drug resistance”, “Pathways in cancer”, and “Cell cycle” KEGG pathways (Fig. 3d). Downregulated miRNAs were involved in pathways including “Apoptosis-multiple species”, “Platinum drug resistance”, and “drug metabolism-other enzymes” (Fig. 3e).

**Table 2:** Significantly up- (*n* = 11) and downregulated (*n* = 15) miRNAs in EVs from serum of patients with leukaemia after irradiation

Mature miRNA ID	Log2FC	Fold Change	Log2(CPM) leukaemia d1	Log2(CPM) leukaemia d0	*p* value	*q* value
*Upregulated (n* *=* *11)*						
Hsa-miR-1277-3p	4.076	16.862	4.112	0.036	0.012	1.000
Hsa-miR-496	3.959	15.551	4.939	0.981	0.004	0.794
**Hsa-miR-548at-5p**	3.896	14.890	3.932	0.036	0.017	1.000
Hsa-miR-1261	3.580	11.960	4.548	0.967	0.012	1.000
Hsa-miR-659-5p	3.550	11.712	3.586	0.036	0.038	1.000
***Hsa-miR-874-3p***	3.438	10.841	5.529	2.091	0.002	0.794
***Hsa-miR-1307-5p***	3.231	9.387	5.913	2.682	0.001	0.653
Hsa-miR-2115-3p	2.825	7.088	3.944	1.119	0.046	1.000
***Hsa-miR-4521***	2.470	5.540	5.648	3.178	0.024	1.000
**Hsa-miR-375-3p**	1.856	3.620	12.328	10.472	0.003	0.794
***Hsa-miR-200b-5p***	1.625	3.084	6.653	5.028	0.029	1.000
*Downregulated (n* *=* *15)*						
**Hsa-miR-216b-5p**	−4.806	0.036	0.036	4.842	0.001	0.653
**Hsa-let-7a-2-3p**	−3.999	0.063	0.036	4.035	0.012	1.000
**Hsa-miR-137-3p**	−3.702	0.077	0.036	3.738	0.021	1.000
Hsa-miR-3182	−3.650	0.080	0.036	3.687	0.025	1.000
Hsa-miR-518e-5p	−3.524	0.087	0.036	3.560	0.031	1.000
Hsa-miR-519a-5p	−3.524	0.087	0.036	3.560	0.031	1.000
Hsa-miR-519b-5p	−3.524	0.087	0.036	3.560	0.031	1.000
Hsa-miR-519c-5p	−3.524	0.087	0.036	3.560	0.031	1.000
Hsa-miR-522-5p	−3.524	0.087	0.036	3.560	0.031	1.000
Hsa-miR-523-5p	−3.524	0.087	0.036	3.560	0.031	1.000
**Hsa-miR-4661-5p**	−3.355	0.098	0.036	3.391	0.047	1.000
Hsa-miR-539-5p	−3.094	0.117	0.662	3.756	0.020	1.000
***Hsa-miR-29a-5p***	−2.620	0.163	3.963	6.583	0.007	1.000
Hsa-miR-4433b-5p	−2.075	0.237	4.135	6.210	0.024	1.000
***Hsa-miR-432-5p***	−1.865	0.275	4.612	6.476	0.022	1.000

*p* value cut-off ≤ 0.05, *q* value cut-off ≤ 1. Mature miRNA ID, ID of mature miRNA; CPM, counts per million reads; log2FC, difference in mean between two groups (Test_CPM vs Control_CPM) of negative binomial random variables; Fold change, 2^test_CPM-control_CPM^; leukaemia d0, patients with leukaemia before irradiation; leukaemia d1, patients with leukaemia after a 2 × 2 Gy irradiation; Log2(CPM), average of log CPM scaled by qCML methods: log2(CPM) of miRNA in respective groups; *p* value, F‑statistic *p* value; *q* value, FDR adjusted *p* value. miRNAs up- or downregulated in the combined cohort and in AML patients are highlighted in bold, while miRNAS up- or downregulated in the combined analysis as well as in ALL single analysis are highlighted in bold italics

### Intersecting miRNAs as biomarkers for leukaemia treatment and irradiation exposure

To further identify miRNAs with a different or common expression in a leukaemia subtype specific manner, we separately analyzed EV’s miRNA cargo content from AML and ALL patients. As depicted in Supplementary Figure S2 the comparison of AML patients with healthy donors yielded 36 upregulated and 15 downregulated miRNAs and 866 mRNAs not differently expressed. The KEGG pathways significantly affected by upregulated miRNAs in AML patients included “Transcriptional misregulation in cancer”, “Hedgehog signaling pathway” and “Acute myeloid leukemia” pathways. Amongst the top regulated KEGG pathways affected by miRNAs being dowregulated, “Osteoclast differentiation”, “p53 signaling pathway” and “FoxO signaling pathway” were evident. By contrast, in ALL patients 47 miRNAs were up- and 17 miRNAs were downregulated while 753 were not altered (Supplementary Figure S3). ALL upregulated miRNAs most significantly covered “Hippo signaling pathway”, “Endocytosis” and “Transcriptional misregulation in cancer”, while downregulated miRNAs were associated with “HIF‑1 signaling”, “MAPK signaling pathway”, and “p53 signaling pathway” like AML patients.

**Fig. 4:**
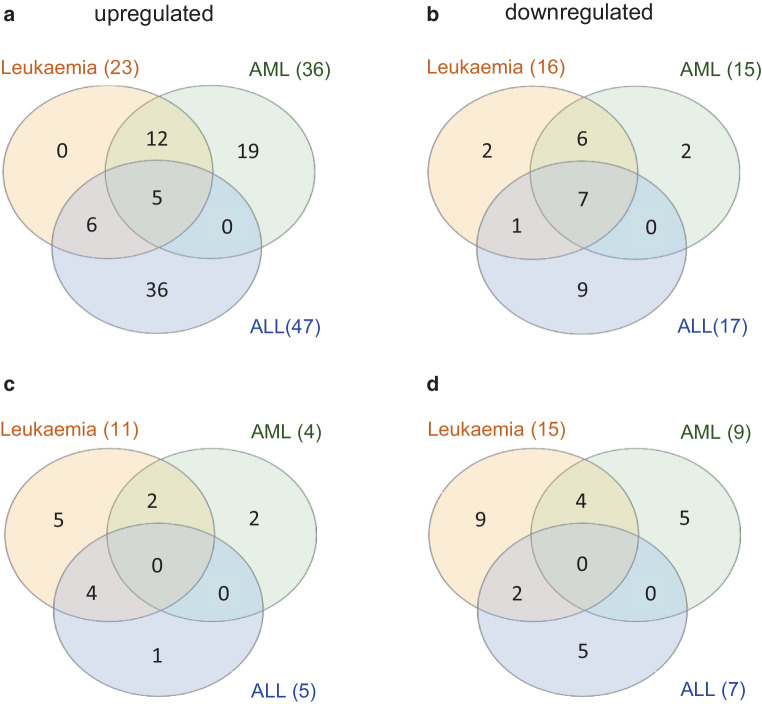
Venn diagram analyses (program InteractiVenn, www.interactivenn.net [19]) of (**a**) upregulated or (**b**) downregulated miRNAs in AML, ALL and combined (AML+ALL = leukaemia) patients and of miRNAs (**c**) upregulated and (**d**) downregulated following TBI in AML, ALL and combined cohort patients (leukaemia)

Venn diagram analyses depicted in Fig. 4a,b specify a total number of 5 miRNAs upregulated in the combined cohort and also in AML and ALL patient single analyses and 7 miRNAs downregulated in all three analyses that might represent robust biomarker signatures for leukaemic diseases. These miRNAs are: hsa-miR-1228-5p, hsa-miR-1469, hsa-miR-576-3p, hsa-miR-1260a, hsa-miR-122-5p in case of upregulated ones, and hsa-miR-16-1-3p, hsa-miR-144-3p, hsa-miR-15b-3p, hsa-miR-144-5p, hsa-miR-184, hsa-miR-142-5p, and hsa-let-7i-5p in case of downregulated miRNAs.

## Discussion

In the last decades, the importance of miRNAs in leukaemic disease initiation and progression and treatment came into light, however, multiple facets in miRNA biology that add complexity to the pathophysiology remain elusive [21]. Moreover, in recent years, EVs have attracted increasing attention as intercellular communicators by delivering their protein, DNA, mRNA and miRNA cargo to recipient cells and by their promising role in providing prognostic and predictive biomarkers [22, 23]. By transferring information between cells in the bone marrow and the stem cell compartment thereby modulating gene expression in the target cells, and by affecting apoptosis and proliferation, EVs also contribute to leukaemia onset, progression and clinical response [7, 8]. In addition, in vitro experiments have shown that irradiation enhances EV production and changes their cargo content [13]. Further in vivo studies revealed that irradiation of donor mice alters miRNA cargo of EVs which in turn contributes to bone marrow dysfunctions in recipient mice [14].

We aimed to investigate alterations in miRNA load in EVs derived from patients with leukaemia before and after irradiation by next generation sequencing. Comparisons of patient’s miRNA cargo with normal donors revealed a differential miRNA load in patients, while TBI (2 × 2 Gy) of patients further modulated the miRNA cargo. Notably, modulations of miRNAs and affected pathways differ in AML and ALL patients (Supplementary Figures S2 and S3) showing a subentity- and treatment-dependent cargo loading. Within the same experimental setup, the comparison of AML patients with healthy donors yielded 36 upregulated miRNAs with KEGG pathways significantly affected to include “Transcriptional misregulation in cancer”, “Hedgehog signaling pathway” and “Acute myeloid leukemia” pathways. Amongst the top regulated pathways affected by downregulated miRNAs “Osteoclast differentiation”, “p53 signaling pathway” and “FoxO signaling pathway” were evident. By contrast, in ALL patients we observed 47 miRNAs (up) and 17 miRNAs (down) differentially regulated with “Hippo signaling pathway”, “Endocytosis” and “Transcriptional misregulation in cancer” pathways affected by upregulated miRNAs. Downregulated miRNAs were associated with “HIF‑1 signaling”, “MAPK signaling pathway”, and “p53 signaling pathway”, similar to AML patients. Thus, differences in miRNA abundances were evident, indicating a leukaemia subtype-dependent regulation and the preference for specific response pathways that may be associated with a different prognosis and response. However, due to the limited number of patients studied for the first time in a parallel approach, a detailed analysis of the different response pathways is not yet considered to be meaningful. Accordingly, we will focus discussion on representative miRNA markers below. Our findings, however, may represent an essential prerequisite for a targeted analysis in an extended cohort of patients.

A recent study from Abdelhamed et al. investigated miRNA load from plasma-derived EVs in 12 recurrent disease patients vs. 12 controls and discovered miR-1246 as a potential biomarker for AML relapse risk and disease burden [24]. The group further developed a miRNA signature with 15 miRNAs upregulated in patients with leukaemia including miR-144-5p, miR-96-5p, miR-145-5p, miR-378a-5p, miR-222-5p, miR-181a-3p, miR-181b-5p, miR-199b-5p, miR-3154, miR-92a-1-5p, miR-25-5p, miR-27a-5p, miR-1246, miR-6503-3p, and miR-6503-5p. Notably, in our analysis, miR-222-5p and miR-27a-5p were confirmed to be upregulated in AML patients, while miR-1246 was significantly upregulated in the ALL patient cohort only. By contrast, miR-144-5p, and miRNA-532 shown to be upregulated in AML by Abdelhamed et al. and by Lin et al. [25] were significantly downregulated in our patient cohorts. Therefore, we consider miR-222-5p and miR-27a-5p to be strong biomarkers for AML disease, while miR-1246 expression requires additional evaluation in extended cohorts of both, AML and ALL patients.

Patients enrolled in our study were treated with preconditioning chemo- and radiation therapy before HCT. We observed leukaemia-associated miRNA species in our cohorts in line with other reports. Notably, the majority of patients displayed MRD [26] or refractory disease that is associated with a poor clinical outcome [27], indicating the presence of low levels of leukaemic blasts that may be considered as sources of disease-associated markers. Differential miRNA-signature, however, may also be associated with the use of different chemotherapeutic drugs and regimes applied in ALL and AML in comparison to healthy controls. By this, findings on chemotherapy-induced EV miRNAs in leukaemia are rare at present, but bone marrow microenvironment stromal cells have been reported to downregulate miR-23a-5p levels in AML cells to protect them from the chemotherapy-induced apoptosis targeting Toll-like receptor 2 expression [28]. In addition, a recent longitudinal single-cell profiling of chemotherapy response in AML stem cells revealed that a low oxidative phosphorylation signature and elevated levels of miR-126 display enforced stemness and quiescence features of these cells [29].

Significant radiation-induced changes of miRNA content in bone marrow-derived EVs from irradiated mice have been reported [14]. Animals were irradiated with a low dose of 0.1 Gy and an intermediate dose of 2 Gy and EVs’ cargo analyses at 24 h revealed a set of eight miRNAs significantly affected in both groups with three up- and five downregulated miRNAs. Among the upregulated markers, the group reported on miR-375-3p, which was also significantly upregulated in our leukaemic patient analysis. An additional report further identified miR-375-3p to be upregulated in serum of irradiated mice at 72 h after irradiation with 7 Gy [30]. Notably, miR-375-3p is shown to be overexpressed in serum of prostate, colorectal and breast cancer patients and to correlate with impaired clinical outcome including tumor progression and metastatic spread [31–33]. Accordingly, these findings confirm miR-375-3p to cover a valid indicator for radiation exposure, both in the murine and human setting. This assumption is further supported by the fact, that miR-375-3p has been identified by bioinformatic in silico analyses to be part of an autophagy related circRNA-miRNA-mRNA-subtypes network (autophagy-related radiosensitivity risk signature) linked to radiation responses and tumor immune microenvironment alterations in lung carcinoma [34].

KEGG pathway analysis of differentially regulated miRNAs in bone marrow-derived murine EVs after radiation exposure, revealed “Wnt and FoxO signaling pathways”, “Protein processing in endoplasmatic reticulum” amongst others, to be affected [14, 35]. In our data sets “Pathways in cancer” (affected in up- and downregulated miRNAs from patients with leukaemia), Wnt pathway (upregulated miRNAs from AML patients), FoxO pathway (downregulated miRNAs from AML patients) and “Protein processing in endoplasmatic reticulum” (upregulated miRNAs from AML patients) were amongst the Top 10 KEGG pathways affected by differential miRNA expression. This indicates these pathways to comprise more common circuits to be associated with radiation-induced miRNA alterations that may provide a starting point for further investigations to unravel mechanisms of the molecular radiation response.

In ALL patients, we observed miR-139-5p to be upregulated in comparison to healthy donors. In that context, a report from Pajic et al. investigated miRNAs with key roles in mediating the clinically relevant processes of radiotherapy resistance in breast cancer [36]. They indicate miR-139-5p as a strong modulator of radiation response by the regulation of genes involved in reactive oxygen species defense and multiple DNA damage repair pathways. The authors further indicated that application of a miR-139-5p mimic to breast cancer cells enhances the radiation response in vitro and in vivo by increasing oxidative stress, induction of apoptosis and accumulation of DNA damage. Although not addressed in the current study, an upregulation of miR-139-5p in ALL patients might render these patients more susceptible to radiation effects.

Our study is subject to some limitations including a small number of patients enrolled, a descriptive character of our findings and due to limitation of material available a lack of confirmation of selected miRNAs by a quantitative PCR approach. These results are, thus, considered preliminary and require validation in an extended cohort of AML and ALL patients. To the best of our knowledge, however, these results, are among the first to compare EVs miRNA cargo load in both, AML and ALL patients that may contribute to a better understanding of leukaemia, treatment response and irradiation-induced modulation of serum miRNA cargo. While we identified an overlap of specific miRNAs up- or downregulated also in the combined analysis and in AML and ALL single analyses, there was no overlap of differentially regulated miRNAs in AML and ALL patients after TBI. In future analyses with more patients, it has to be confirmed whether these differences depend on the low patient numbers or whether the radiation-induced regulation of miRNA levels after TBI should be studied separately for each leukaemia type. In summary, we consider the potential of EV-derived miRNAs to fulfill the role of future biomarkers in patients with leukaemia subjected to consolidation/preconditioning chemotherapy and TBI.

## Supplementary Information

**Supplementary Figure S1.** Workflow of miRNA sequencing data analysis (ArrayStar Inc.).

**Supplementary Figure S2.** Differentially expressed miRNAs of EVs derived from serum of AML patients before irradiation vs. healthy donors. (A) Volcano plot, (B) heatmap of upregulated and (C) heatmap of downregulated miRNAs in EVs from serum of AML patients. (D) Top 10 significant KEGG pathways affected by upregulated miRNAs of AML patients vs. healthy donors, ordered from top to bottom by *p*-value. (E) Top 10 significant KEGG pathways affected by downregulated miRNAs of AML patients vs. healthy donors.

**Supplementary Figure S3**. Differentially expressed miRNAs of EVs derived from serum of ALL patients before irradiation vs. healthy donors. (A) Volcano plot, (B) heatmap of upregulated and (C) heatmap of downregulated miRNAs in ALL patients’ EVs. (D) Top 10 KEGG pathways affected by upregulated miRNAs of ALL patients vs. healthy donors, ordered from top to bottom by *p*-value. (E) Top 10 significant KEGG pathways affected by downregulated miRNAs of ALL patients vs. healthy donors.

**Supplementary Figure S4**. Differentially expressed miRNAs after irradiation of AML patients. (A) Volcano plot, (B) heatmap of upregulated and (C) heatmap of downregulated miRNAs after irradiation of AML patients. (D) Top 10 KEGG pathways affected by upregulated miRNAs or (E) downregulated miRNAs from irradiated vs. non-irradiated AML patients.

**Supplementary Figure S5**. Differentially expressed miRNAs in EVs derived from serum of ALL patients after irradiation vs. before irradiation. (A) Volcano plot, (B) heatmap of upregulated and (C) heatmap of downregulated miRNAs after irradiation of ALL patients. (D) Top 10 KEGG pathways affected by upregulated miRNAs or (E) downregulated miRNAs in serum EVs from irradiated vs. non-irradiated ALL patients.
