# Supplementary material for: Irradiation alters extracellular vesicle microRNA load in the serum of patients with leukaemia
Source: Strahlenther Onkol. 2024 Sep 26;201(2):173–84. doi: 10.1007/s00066-024-02307-6 (PMC11754379; doi:10.1007/s00066-024-02307-6)
Supplement: Supplementary file 1 — Supplementary Figure S1. Workflow of miRNA sequencing data analysis (ArrayStar Inc.). [file 66_2024_2307_MOESM1_ESM.pptx]

## Slide 1
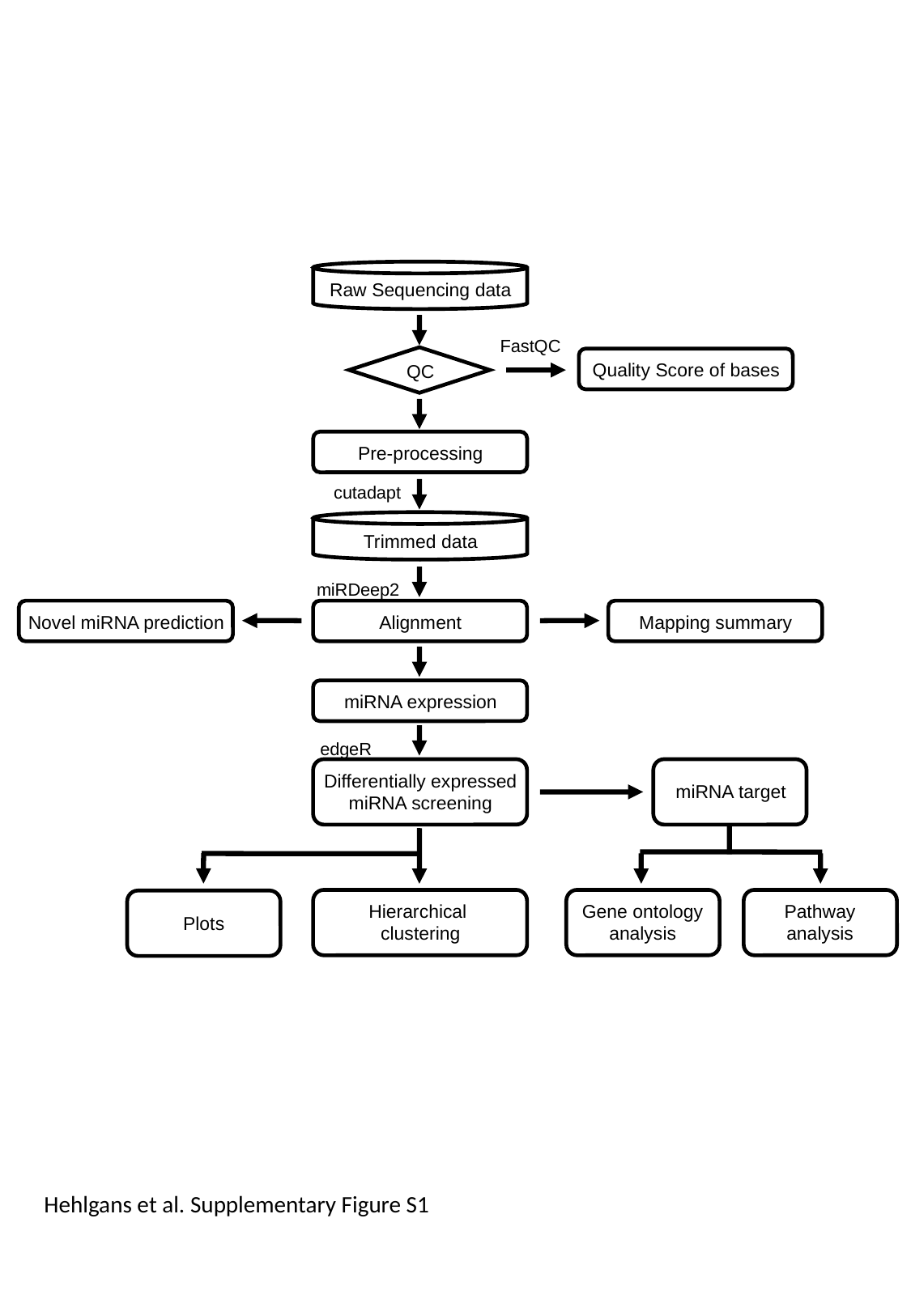

Raw Sequencing data
FastQC
Quality Score of bases
QC
Pre-processing
cutadapt
Trimmed data
miRDeep2
Novel miRNA prediction
Alignment
Mapping summary
miRNA expression
edgeR
Differentially expressed miRNA screening
miRNA target
Hierarchical
clustering
Gene ontology
analysis
Pathway
analysis
Plots
Hehlgans et al. Supplementary Figure S1
