# Supplementary material for: Irradiation alters extracellular vesicle microRNA load in the serum of patients with leukaemia
Source: Strahlenther Onkol. 2024 Sep 26;201(2):173–84. doi: 10.1007/s00066-024-02307-6 (PMC11754379; doi:10.1007/s00066-024-02307-6)
Supplement: Supplementary file 2 — Supplementary Figure S2. Differentially expressed miRNAs of EVs derived from serum of AML patients before irradiation vs. healthy donors. (A) Volcano plot, (B) heatmap of upregulated and (C) heatmap of downregulated miRNAs in EVs from serum of AML patients. (D) Top 10 significant KEGG pathways affected by upregulated miRNAs of AML patients vs. healthy donors, ordered from top to bottom by p-value. (E) Top 10 significant KEGG pathways affected by downregulated miRNAs of AML patients vs. healthy donors. [file 66_2024_2307_MOESM2_ESM.pptx]

## Slide 1
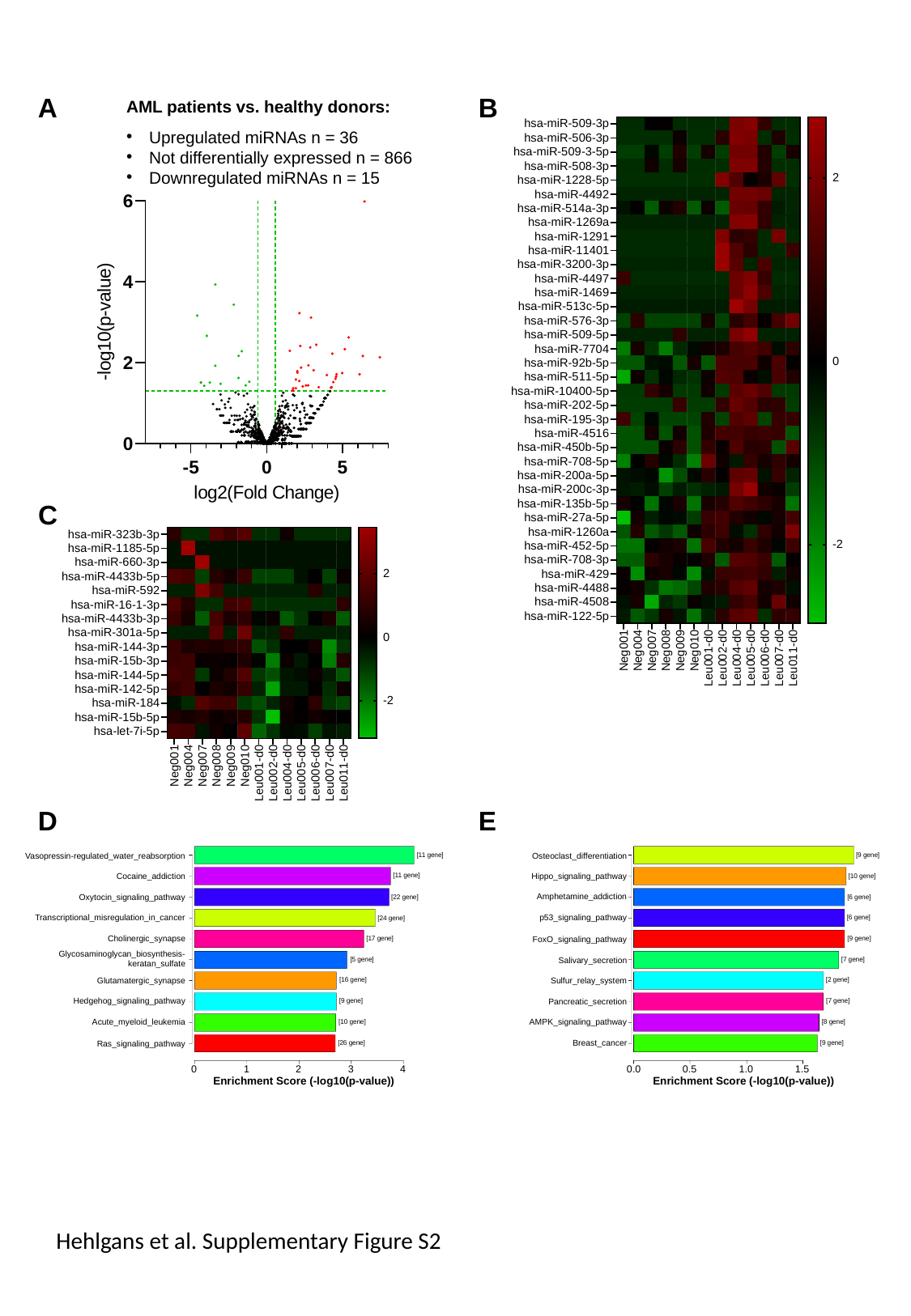

A
B
AML patients vs. healthy donors:
Upregulated miRNAs n = 36
Not differentially expressed n = 866
Downregulated miRNAs n = 15
C
D
E
Vasopressin-regulated_water_reabsorption
Osteoclast_differentiation
[11 gene]
[11 gene]
[22 gene]
[24 gene]
[17 gene]
[5 gene]
[16 gene]
[9 gene]
[10 gene]
[26 gene]
[9 gene]
[10 gene]
[6 gene]
[6 gene]
[9 gene]
[7 gene]
[2 gene]
[7 gene]
[8 gene]
[9 gene]
Cocaine_addiction
Hippo_signaling_pathway
Amphetamine_addiction
Oxytocin_signaling_pathway
Transcriptional_misregulation_in_cancer
p53_signaling_pathway
Cholinergic_synapse
FoxO_signaling_pathway
Glycosaminoglycan_biosynthesis-keratan_sulfate
Salivary_secretion
Glutamatergic_synapse
Sulfur_relay_system
Hedgehog_signaling_pathway
Pancreatic_secretion
Acute_myeloid_leukemia
AMPK_signaling_pathway
Breast_cancer
Ras_signaling_pathway
0
1
2
3
4
0.0
0.5
1.0
1.5
Enrichment Score (-log10(p-value))
Enrichment Score (-log10(p-value))
Hehlgans et al. Supplementary Figure S2
