# Supplementary material for: Irradiation alters extracellular vesicle microRNA load in the serum of patients with leukaemia
Source: Strahlenther Onkol. 2024 Sep 26;201(2):173–84. doi: 10.1007/s00066-024-02307-6 (PMC11754379; doi:10.1007/s00066-024-02307-6)
Supplement: Supplementary file 3 — Supplementary Figure S3. Differentially expressed miRNAs of EVs derived from serum of ALL patients before irradiation vs. healthy donors. (A) Volcano plot, (B) heatmap of upregulated and (C) heatmap of downregulated miRNAs in ALL patients’ EVs. (D) Top 10 KEGG pathways affected by upregulated miRNAs of ALL patients vs. healthy donors, ordered from top to bottom by p-value. (E) Top 10 significant KEGG pathways affected by downregulated miRNAs of ALL patients vs. healthy donors. [file 66_2024_2307_MOESM3_ESM.pptx]

## Slide 1
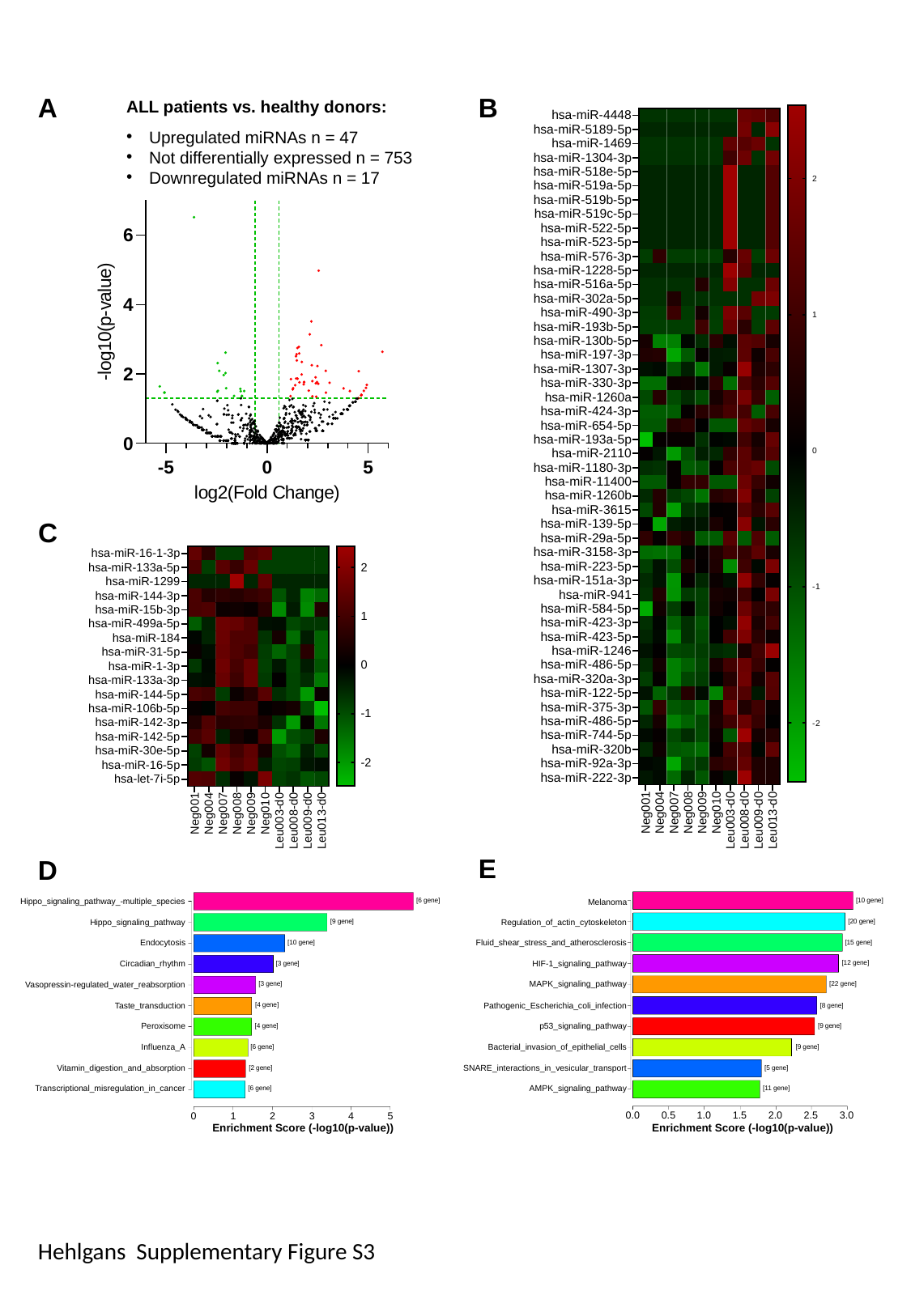

A
B
ALL patients vs. healthy donors:
Upregulated miRNAs n = 47
Not differentially expressed n = 753
Downregulated miRNAs n = 17
C
E
D
Hippo_signaling_pathway_-multiple_species
[6 gene]
[9 gene]
[10 gene]
[3 gene]
[3 gene]
[4 gene]
[4 gene]
[6 gene]
[2 gene]
[6 gene]
[10 gene]
[20 gene]
[15 gene]
[12 gene]
[22 gene]
[8 gene]
[9 gene]
[9 gene]
[5 gene]
[11 gene]
Melanoma
Hippo_signaling_pathway
Regulation_of_actin_cytoskeleton
Endocytosis
Fluid_shear_stress_and_atherosclerosis
Circadian_rhythm
HIF-1_signaling_pathway
MAPK_signaling_pathway
Vasopressin-regulated_water_reabsorption
Taste_transduction
Pathogenic_Escherichia_coli_infection
Peroxisome
p53_signaling_pathway
Bacterial_invasion_of_epithelial_cells
Influenza_A
SNARE_interactions_in_vesicular_transport
Vitamin_digestion_and_absorption
Transcriptional_misregulation_in_cancer
AMPK_signaling_pathway
0.0
0.5
1.0
1.5
2.0
2.5
3.0
0
1
2
3
4
5
Enrichment Score (-log10(p-value))
Enrichment Score (-log10(p-value))
Hehlgans Supplementary Figure S3
