# Supplementary material for: Irradiation alters extracellular vesicle microRNA load in the serum of patients with leukaemia
Source: Strahlenther Onkol. 2024 Sep 26;201(2):173–84. doi: 10.1007/s00066-024-02307-6 (PMC11754379; doi:10.1007/s00066-024-02307-6)
Supplement: Supplementary file 4 — Supplementary Figure S4. Differentially expressed miRNAs after irradiation of AML patients. (A) Volcano plot, (B) heatmap of upregulated and (C) heatmap of downregulated miRNAs after irradiation of AML patients. (D) Top 10 KEGG pathways affected by upregulated miRNAs or (E) downregulated miRNAs from irradiated vs. non-irradiated AML patients. [file 66_2024_2307_MOESM4_ESM.pptx]

## Slide 1
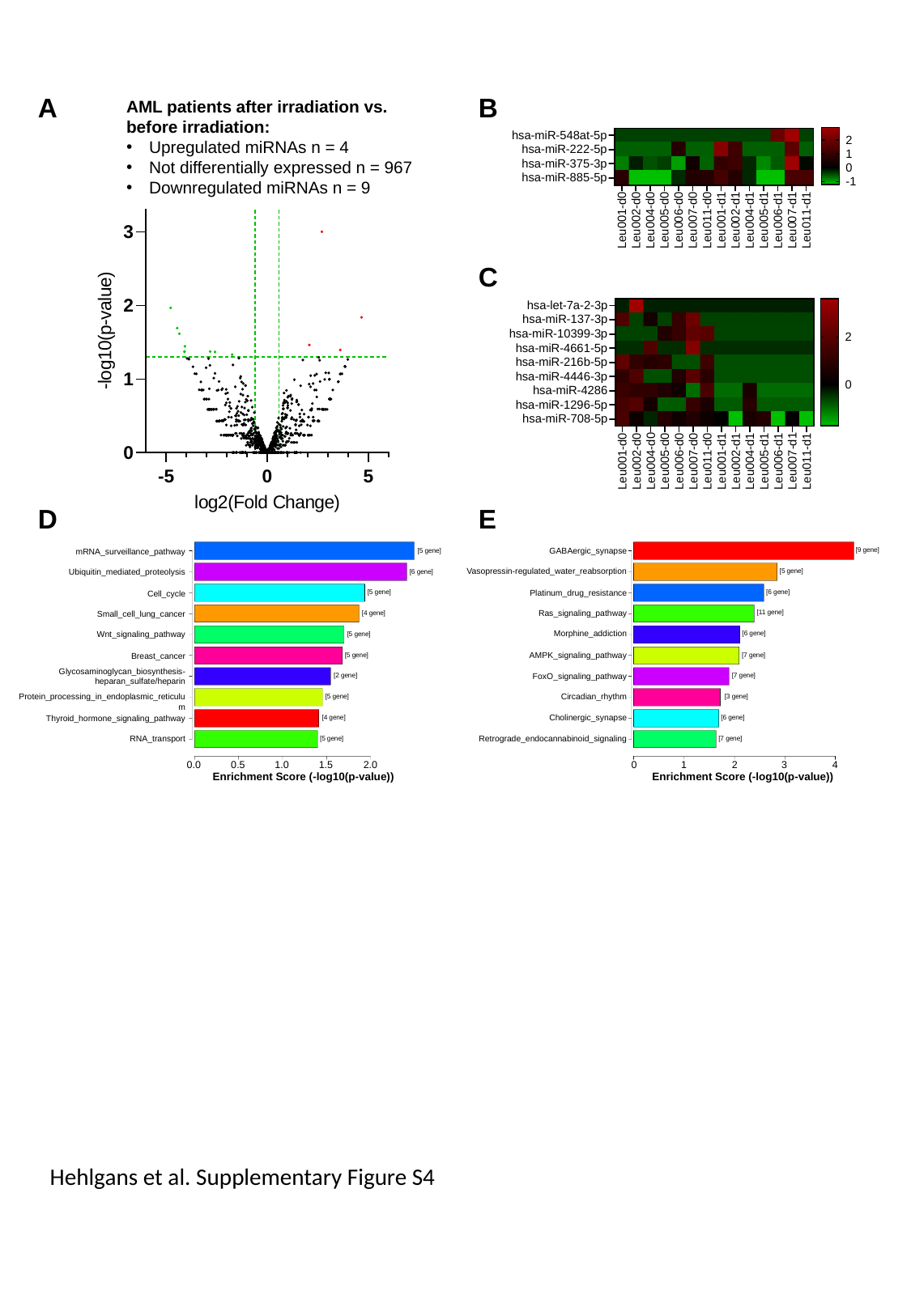

A
B
AML patients after irradiation vs. before irradiation:
Upregulated miRNAs n = 4
Not differentially expressed n = 967
Downregulated miRNAs n = 9
C
D
E
GABAergic_synapse
[9 gene]
[5 gene]
[6 gene]
[11 gene]
[6 gene]
[7 gene]
[7 gene]
[3 gene]
[6 gene]
[7 gene]
[5 gene]
[6 gene]
[5 gene]
[4 gene]
[5 gene]
[5 gene]
[2 gene]
[5 gene]
[4 gene]
[5 gene]
mRNA_surveillance_pathway
Vasopressin-regulated_water_reabsorption
Ubiquitin_mediated_proteolysis
Platinum_drug_resistance
Cell_cycle
Ras_signaling_pathway
Small_cell_lung_cancer
Morphine_addiction
Wnt_signaling_pathway
AMPK_signaling_pathway
Breast_cancer
Glycosaminoglycan_biosynthesis-heparan_sulfate/heparin
FoxO_signaling_pathway
Protein_processing_in_endoplasmic_reticulum
Circadian_rhythm
Cholinergic_synapse
Thyroid_hormone_signaling_pathway
Retrograde_endocannabinoid_signaling
RNA_transport
0.0
0.5
1.0
1.5
2.0
0
1
2
3
4
Enrichment Score (-log10(p-value))
Enrichment Score (-log10(p-value))
Hehlgans et al. Supplementary Figure S4
