# Supplementary material for: Irradiation alters extracellular vesicle microRNA load in the serum of patients with leukaemia
Source: Strahlenther Onkol. 2024 Sep 26;201(2):173–84. doi: 10.1007/s00066-024-02307-6 (PMC11754379; doi:10.1007/s00066-024-02307-6)
Supplement: Supplementary file 5 — Supplementary Figure S5. Differentially expressed miRNAs in EVs derived from serum of ALL patients after irradiation vs. before irradiation. (A) Volcano plot, (B) heatmap of upregulated and (C) heatmap of downregulated miRNAs after irradiation of ALL patients. (D) Top 10 KEGG pathways affected by upregulated miRNAs or (E) downregulated miRNAs in serum EVs from irradiated vs. non-irradiated ALL patients. [file 66_2024_2307_MOESM5_ESM.pptx]

## Slide 1
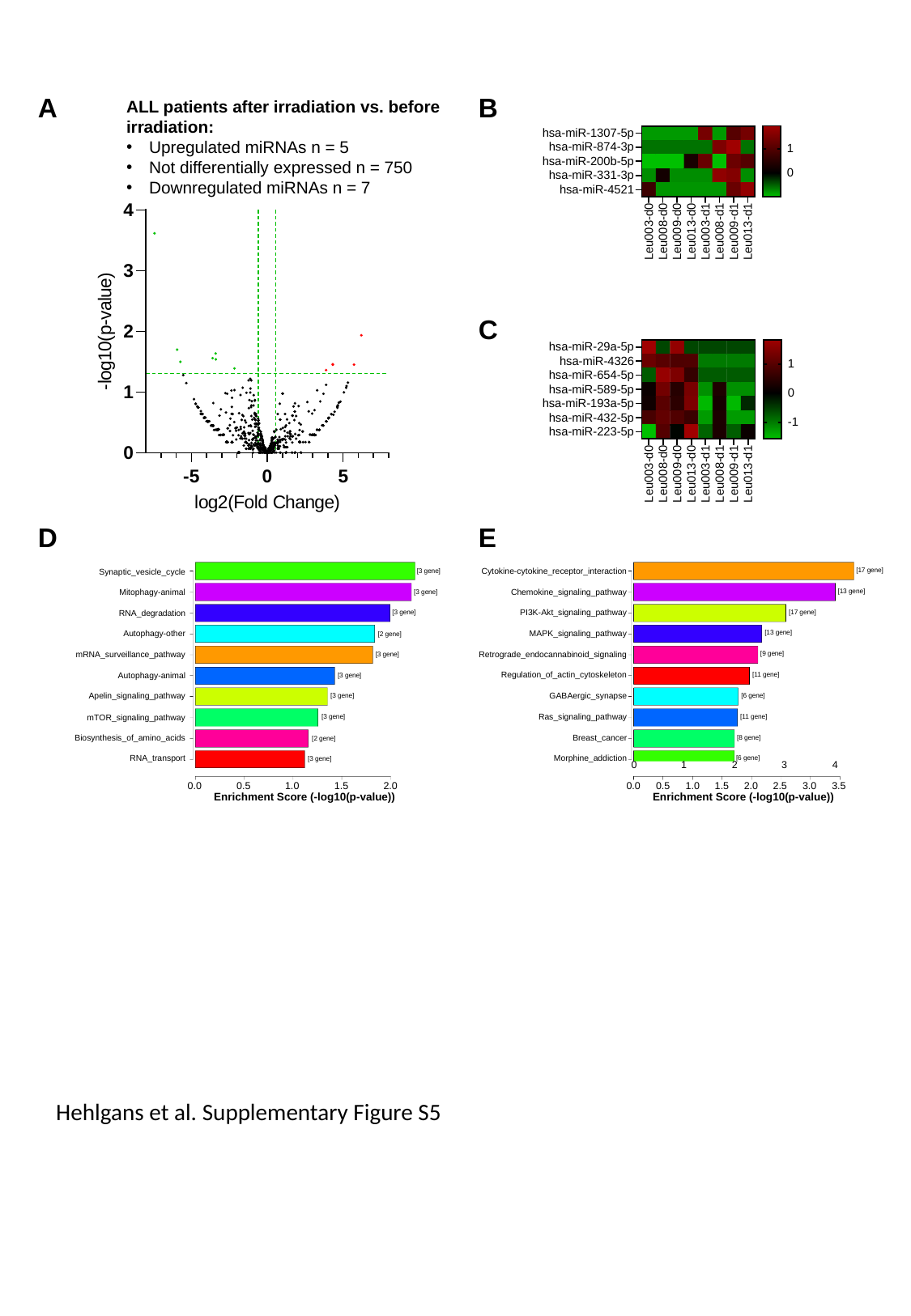

A
B
ALL patients after irradiation vs. before irradiation:
Upregulated miRNAs n = 5
Not differentially expressed n = 750
Downregulated miRNAs n = 7
C
D
E
Cytokine-cytokine_receptor_interaction
[17 gene]
[13 gene]
[17 gene]
[13 gene]
[9 gene]
[11 gene]
[6 gene]
[11 gene]
[8 gene]
[6 gene]
Synaptic_vesicle_cycle
[3 gene]
[3 gene]
[3 gene]
[2 gene]
[3 gene]
[3 gene]
[3 gene]
[3 gene]
[2 gene]
[3 gene]
Chemokine_signaling_pathway
Mitophagy-animal
PI3K-Akt_signaling_pathway
RNA_degradation
MAPK_signaling_pathway
Autophagy-other
Retrograde_endocannabinoid_signaling
mRNA_surveillance_pathway
Regulation_of_actin_cytoskeleton
Autophagy-animal
GABAergic_synapse
Apelin_signaling_pathway
Ras_signaling_pathway
mTOR_signaling_pathway
Breast_cancer
Biosynthesis_of_amino_acids
RNA_transport
Morphine_addiction
0
1
2
3
4
0.0
0.5
1.0
1.5
2.0
0.0
0.5
1.0
1.5
2.0
2.5
3.0
3.5
Enrichment Score (-log10(p-value))
Enrichment Score (-log10(p-value))
Hehlgans et al. Supplementary Figure S5
